# Nuclear ribosomal internal transcribed spacer 1 (ITS1) variation in the *Anastrepha fraterculus* cryptic species complex (Diptera, Tephritidae) of the Andean region

**DOI:** 10.3897/zookeys.540.6147

**Published:** 2015-11-26

**Authors:** Bruce D. Sutton, Gary J. Steck, Allen L. Norrbom, Erick J. Rodriguez, Pratibha Srivastava, Norma Nolazco Alvarado, Fredy Colque, Erick Yábar Landa, Juan José Lagrava Sánchez, Elizabeth Quisberth, Emilio Arévalo Peñaranda, P. A. Rodriguez Clavijo, Jeniffer K. Alvarez-Baca, Tito Guevara Zapata, Patricio Ponce

**Affiliations:** 1 Division of Plant Industry, Florida Department of Agriculture and Consumer Services, Gainesville, FL, USA; 2 Systematic Entomology Laboratory, United States Department of Agriculture, Washington, D.C., USA; 3 Servicio Nacional de Sanidad Agraria del Perú, Lima, Perú; 4 Servicio Nacional de Sanidad Agropecuaria e Inocuidad Alimentaria, Bolivia; 5 Universidad Nacional de San Antonio Abad del Cusco, Cusco, Perú; 6 Direccion de Sanidad Agroalimentaria, Gobierno Autónomo Departamental Santa Cruz, Santa Cruz, Bolivia; 7 Instituto Colombiano Agropecuario, Bogota, Colombia; 8 Millennium Nucleus Centre in Molecular Ecology and Evolutionary Applications in the Agroecosystems, 2 Norte 685, Talca, Chile; 9 Centro de Investigacion Translaciónal, La Universidad de las Americas, Quito, Ecuador

**Keywords:** Molecular, fruit fly, Diptera

## Abstract

The nuclear ribosomal internal transcribed spacer 1 (ITS1) was sequenced for *Anastrepha fraterculus* (Wiedemann, 1830) originating from 85 collections from the northern and central Andean countries of South America including Argentina (Tucumán), Bolivia, Perú, Ecuador, Colombia, and Venezuela. The ITS1 regions of additional specimens (17 collections) from Central America (México, Guatemala, Costa Rica, and Panamá), Brazil, Caribbean Colombia, and coastal Venezuela were sequenced and together with published sequences (Paraguay) provided context for interpretation. A total of six ITS1 sequence variants were recognized in the Andean region comprising four groups. Type I predominates in the southernmost range of *Anastrepha fraterculus*. Type II predominates in its northernmost range. In the central and northern Andes, the geographic distributions overlap and interdigitate with a strong elevational effect. A discussion of relationships between observed ITS1 types and morphometric types is included.

internal transcribed spacer 1

## Introduction

The *Anastrepha* fauna of the central and northern Andes and Andean periphery is extremely rich, but incompletely known, and includes the poorly understood Andean populations of the serious economic pest species *Anastrepha fraterculus* (Wiedemann), which is interpreted as a cryptic species complex as recently reviewed by Hernández-Ortiz et al. (2012, 2015). The taxonomic structure of the *Anastrepha fraterculus* cryptic species complex remains inadequately resolved, even as to the number of species involved and their distributional patterns. As part of an ongoing project to improve the identification of *Anastrepha* species, an effort to better resolve and understand the taxonomic structure of this complex was undertaken utilizing sequence analysis of select mitochondrial and nuclear gene regions, with emphasis on the fauna of the Andean region.

Preliminary Sanger sequencing of potentially useful DNA regions for a subset of *Anastrepha fraterculus* specimens from two sites in the central Andes of Peru representing “low” and “high” elevation populations (Peru colony, Lima, (n=5) and Cusco, Calca, (n=5) respectively) included the mitochondrial cytochrome oxidase I (COI, in part, including the Folmer fragment) and ribosomal r16S (in part), and the nuclear ribosomal r18S (in part), internal transcribed spacer (ITS, complete), and r28S (in part, including the expansion domains D1-D8). It also included the nuclear protein coding genes elongation factor 1 (EF1∝, in part) and the carbamoyl phosphate synthetase domain (CAD, intron1-exon1, in part). These were also sequenced for other *Anastrepha fraterculus* species group taxa including *Anastrepha suspensa* (Loew), *Anastrepha distincta* Greene, *Anastrepha ludens* (Loew), and *Anastrepha obliqua* (Macquart).

This initial survey established that taxonomically informative and diagnostic sequence polymorphism was present in the ribosomal internal transcribed spacer 1 (ITS1). Further sequence analysis then concentrated on ITS1 and geographical coverage was expanded to include the entire project region. We here report ITS1 sequence polymorphism patterns, their geographical correlations, and taxonomic implications for Andean populations of *Anastrepha fraterculus*.

## Methods

The majority of specimens in this study were adult females. Taxonomic determinations as *Anastrepha fraterculus* were based on adult morphological criteria following Norrbom et al. (2012 onwards). After removal of tissue for DNA analysis specimens were deposited in the FSCA as vouchers.

The geographical region chosen for the initial stage of this project included the central and northern Andes and Andean periphery of Bolivia, Perú, Ecuador, Colombia, Venezuela, and Argentina. For comparison, limited numbers of specimens were also available from outside of the core region including México, Guatemala, Costa Rica, Caribbean Colombia (Isla de San Andrés), southern and southeastern Brazil, and coastal Venezuela (Los Caracas), as well as ITS1 sequences (NCBI) derived from *Anastrepha fraterculus* originating from localities in Paraguay (Lopes 2010, Lopes et al. 2013).

Most specimens originated from McPhail-type fruit fly traps using yeast hydrolysate bait with, or without, borate preservative. Lesser numbers were collected using multi-lure traps (ML) or reared from host fruits. Trapped or reared specimens were stored in alcohol (70–100% isopropanol or ethanol); or in the case of some archival FSCA samples, stored frozen at -80 °C from as early as 1988.

Acronyms for organizations that provided specimens or DNA sequences are as follows:

DSA Dirección de Sanidad Agroalimentaria, Gobierno Autónomo Departamental Santa Cruz, Bolivia.

FSCA Florida State Collection of Arthropods, Division of Plant Industry, Florida Department of Agriculture and Consumer Services, United States of America.

IAEA, IPCL International Atomic Energy Agency, Insect Pest Control Laboratory, Austria.

ICA Instituto Colombiano Agropecuario, Colombia.

NCBI National Center for Biotechnology Information, United States of America.

SENASA Servicio Nacional de Sanidad Agraria del Perú, Perú.

SENASAG Servicio Nacional de Sanidad Agropecuaria e Inocuidad Alimentaria, Bolivia.

USDA, ARS United States Department of Agriculture, Agricultural Research Service, United States of America.

**Figure 1.**
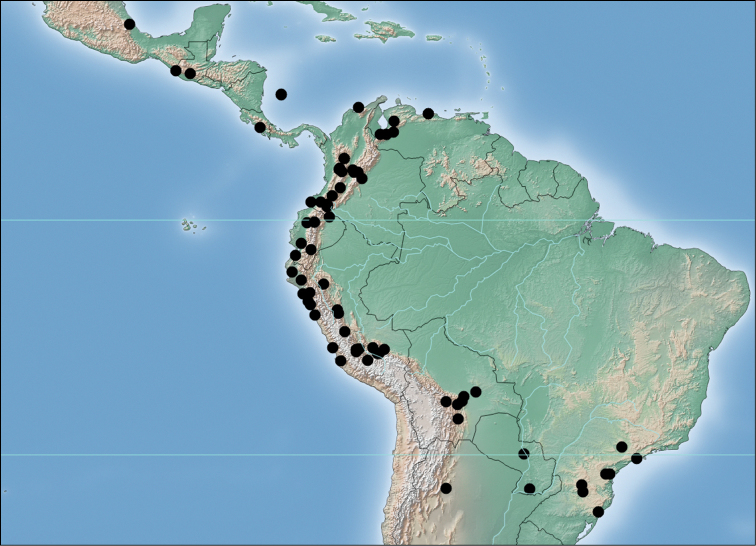
*Anastrepha fraterculus* collection localities.

ITS1 sequences were constructed for 299 *Anastrepha fraterculus* specimens representing 85 collections (Figure 1) from the central and northern Andes including Perú (n=119; SENASA, FSCA, IAEA-IPCL, this project), Bolivia (n=28; SENASAG, DSA, FSCA, this project), Colombia (n=125; ICA, IAEA-IPCL, this project), Ecuador (n=14; FSCA, this project), Venezuela (n=7; FSCA), and Argentina (n=6; IAEA-IPCL).

To help place geographical distributions and taxonomic identities in context, specimens of *Anastrepha fraterculus* from localities outside of the Andean region having identical ITS1 sequences to those recorded from the Andean localities were also included in this analysis. The latter represented 17 collections (n=44) from Mesoamerica, including México (n=3; FSCA, IAEA-IPCL), Guatemala (n=4; FSCA), and Costa Rica (n=2; FSCA), Brazil (n=26; FSCA, IAEA-IPCL, USDA-ARS), Colombia (n=8; ICA) and Venezuela (n=1; FSCA).

Paraguay was represented by ITS1 sequences deposited in the NCBI (HQ829865 to HQ829879) (Lopes 2010, Lopes et al. 2013).

Each collection listed below is described by country, collection number, locality, geographical coordinates (latitude and longitude) when provided, date, collector, other relevant label data, number of specimens, and sex (male=M, female=F, unknown=?): **ARGENTINA**: 1) **Tucumán**, colony 35 in Vienna, Austria, April 2004, IAEA-IPCL, 6 F. **BOLIVIA**: 2) **Chuquisaca**: Surema, 01-06-02-19, 15 June 2010, SENASAG, 2 F. 3) **Santa Cruz**: Refugio Los Volcanes, 63.60W 18.11S, 1040-1050m, reared ex fruit of *Pouteria glomerata* (Miq.) Radik, 20-24 October 2013, A.L. Norrbom & B.D. Sutton, 8 F; 4) Refugio Los Volcanes, 63.60W 18.11S, 1040-1050 m, reared ex fruit of *Myrciaria floribunda* (H. West ex Willd.) Berg., A.L. Norrbom & B.D. Sutton, 8 F; 5) Totaí, 62.2494W 17.20322S, 260 m, 19 August 2014, SENASAG, 6 F; 6) Totaí, 62.2494W 17.20322S, 260 m, 5 August 2014, SENASAG, 1 F; 7) Valle Grande, McPhail Trap, 13 September 2001, DSA, 3 F. **BRAZIL**: 8) **Paraná**: São João de Graciosa, Poço Preto, 48.86842W 25.39829S, 76m, reared ex fruit of *Psidium guajava* L., 28 February 2015, M. Savaris & A.L. Norrbom, 4 F; 9) Palmas, Linha Algeria, Fazenda Cerro Chato, 51.67301W 26.50278S, 1208 m, butterfly trap, 2-4 March 2015, Sepulveda, 1 F. 10) **Rio Grande do Sul**: Vacaria, colony 36 in Vienna, Austria, May 2010, IAEA-IPCL, 2 larvae. 11) **São Paulo**: Piracicaba: colony 37 in Vienna, Austria, February 2011, IAEA-IPCL, 10 F; 12) Bertioga, sea level, reared ex *Terminalia catappa* L., 2 F; 13) Itaquera, reared ex guava, 25 July 1989, Malavasi & Morgante, 2 F. 14) **Santa Catarina**: Friburgo, reared ex Fejoa (*Acca sellowiana* (O. Berg) Burret), 2 F; 15) Joacaba, reared ex guava, 2 F; 16) P. N. Aparados da Serra, Linha Rio do Boi, 50.00469W 29.20229S, 208 m, butterfly trap, 6-9 March 2015, T. Sepulveda, 1 F. **COLOMBIA**: 17) **Antioquia**: Rionegro, Vereda Barro Blanco, 75.40494W 6.15378N, 2144 m, ML Trap, 11 July 2013, A. Florez (ICA), 8 F; 18) Rionegro, 75.4155W 6.1303N, 2151 m, ML Trap, 03 August 2010, E. Arévalo (ICA), 8 F; 19) Rionegro, Aeropuerto MC, El Platanal, 75.43533W 6.17496N, 2174 m, ML Trap, 30 May 2013, A. Florez (ICA), 8 F. 20) **Cauca**: Timbio, 76.63139W 2.42296N, 1752 m, ruta 415300007, 18 July 2014, Z. Vargas, 7 F. 21) **Cundinamarca**: Anolaima, Finca Villa Mariana, 74.47569W 4.80175N, 1558 m, ex *Coffea arabica* L., 16 September 2014, E.J. Rodriguez & P.A. Rodriguez, 2 larvae; 22) same location, multilure trap, 07 September 2014, E.J. Rodriguez & P.A. Rodriguez, 2 F. 23) Guaduas, campo de tejo, 74.55471W 5.03767N, 1668 m, 23 August 2014, P. Correa, 8 F. 24) **Huila**: Palestina, 76.1390W 1.7319N, 1531m, ruta 415300007, 21 February 2014, ICA, 2 F; 25) Palestina, 70.1726W 1.7033N, 1798m, ruta 415300007, 21 February 2014, ICA, 1 F; 26) Palestina, 76.1351W 1.7099N, 1769m, ruta 415300007, 20 February 2014, (ICA), 1 F; 27) Palestina, 76.1683W 1.7070N, 1790m, ruta 415300007, 21 February 2014, ICA, 1 F; 28) Palestina, 70.1174W 1.7175N, 1511m, ruta 415300007, 20 February 2014, ICA, 3 F; 29) **Magdalena**: Santa Martha, 73.9959W 11.28432N, 153 m, 09 May 2014, J. Amezquita, 1 F; 30) Santa Marta, 73.77654W 11.24910N, 46m, 12 August 2014, J. Amezquita, 1 F. 31) **Meta**: Lejanias, Predio el Vergel, 73.9073W 3.4556N, 482m, 15 June 2014, ICA, 1 F; 32) Lejanias, predio Buenos Aires, 73.9006W 3.4612N, 475m, 16 June 2014, ICA, 1 F. 33) **Nariño**: Tumaco, Kiosco Panorama, 78.73395W 1.82822N 8.7 m, ICA, 2 F; 34) Tumaco, Estrella del Mar, 78.75569W 1.81244N, 13.4 m, ICA, 8 F; 35) Arboleda, Los Balsos, 77.0964W 1.44708N, 1900 m, ICA, 9 F; 36) Buesaco, El Nispero, 77.16837W 1.36098N, 2007 m, ICA, 3 F; 37) La Union, Chaguarurco, 77.16488W 1.59419N, 1849 m, ICA, 4 F. 38) Pasto, Confrut El Eden, 77.27045W 1.19534N, 2562 m, ICA, 5 F. 39) **Putumayo**: Predio Rivera la Bomba, 76.91229W 0.33237N, 332 m, 15 March 2005, F. Cespedes, 8 F. 40) **Risaralda**: Apia, Via Apia Belen, 75.87456W 5.18857N, 1413 m, 12 August 2014, D. Garcia, 8 F; 41) Santuario, predio la Marina, 75.94676W 5.05153N, 1106 m, 04 June 2014, D. Gómez, 8 F; 42) Dos Quebradas, predio San Isidro, 75.66122W 4.84610N, 1497 m, 06 March 2014, D. Gomez, 8 F. 43) **San Andrés y Providencia**: Orange Hill, 814.7085W 12.5584N, 8m, 26 December 2013, ICA, 5 F; 44) Arlis Williams, 81.7086W 12.5584N, 0m, 29 November 2013, ICA, 3 F. 45) **Tolima**: Vereda de Gamboa, 4.40909W 75.33638N, 1635m, colony in Vienna, Austria, IAEA-IPCL, 2 F; 46) Ibagué/lavado, colony 40 in Vienna, Austria, July 2010, IAEA-IPCL, 5 F. **COSTA RICA**: 47) Dominical, sea level, reared ex *Terminalia catappa*, March 1989, M. Condon, 2 F. **ECUADOR**: 48) **Azuay**: Gualaceo, ca. 10km s.e., reared ex peach, 10 March 1994, G.J. Steck & B. McPheron, 2 ?. 49) **Guayas**: Taura, 100m, McPhail in mango, 28 January 2001, P. Ponce, 6 F. 50) **Pichincha**: Tubaco, Expt. Stn., ex toronja blanca, 24 February 1994, J. Vilatuna, 4 ?. 51) **Tungurahua**: Patate, 2000 m, McPhail in *Psidium guajava*, 22 October 2000, P. Ponce, 2 F. **GUATEMALA**: 52) Chim., ex guava, November 1989, 2 F; 53) Chim., ex guava, November 1989, 2 F. **MEXICO**: 54) **Chiapas**: Tapachula, Metapa, ex guava, March 1989, 1 F. 55) **Veracruz**: Xalapa, colony 39 in Vienna, Austria, August 2010, IAEA-IPCL, 2 F. **PERÚ**: 56) **Amazonas**: Huayabamba, Huambo, Nuevo Horizonte, 17 August 2006, SENASA, 1 F; 57) Huayabamba, Longar, Michina, 21 September 2006, SENASA, 2 F; 58) Huayabamba, Huambo, Nuevo Horizonte, 21 September 2006, SENASA, 3 F. 59) **Ancash**: Casma, Urbano, Calles, 31 August 2006, SENASA, 9 F; 60) Casma, Urbano, Avenidas, 23 August 2006, SENASA, 3 F; 61) Casma, Urbano, Calles, 23 August 2006, SENASA, 8 F; 62) **Apurimac**: 23 June 2005, SENASA, 2 F. 63) **Ayacucho**: Huamanga, Huatatas, Huatatas, 06 December 2005, SENASA, 1 F; 64) Huamanga, Pongora-Chacco, Muyurina, 06 December 2005, SENASA, 1 F; 65) Huamanga, Yucfaes, Pucahuasi, 07 December 2005, SENASA, 2 F; 66) Huanta, Urbano, Huanta, 13 December 2005, SENASA, 3 F. 67) **Cajamarca**: Alto Jequetepeque, Alto Jequetepeque, 05 July 2006, SENASA, 1 F. 68) **Cusco**: colony in Vienna, Austria, IAEA–IPCL, 2 F. 69) Echarati, Chontachayoc, 19 January 2014, T. Guevara, 8 F. 70) Urubamba, Calca, Sillacancha, 29 September 2006, SENASA, 2 F. 71) Pilcopata, 71.41946W 12.89219S, 559m, 1 March-12 April 2013, M. Choque, 1 F. 72) **Huánuco**: Huallaga-Central, Aucayacu, Santa Lucia, 17 October 2006, SENASA, 3 F; 73) Huallaga-Central, Aucayacu, Pucayacu, 01 November 2006, SENASA, 3 F; 74) Huallaga-Central, Aucayacu, Pueblo Nuevo, 31 October 2006, SENASA, 1 F; 75) Huánuco: Huallaga-Central, Aucayacu, Pueblo Nuevo, 18 July 2006, SENASA, 5 F; 76) Huallaga-Central, Aucayacu, Pueblo Nuevo, SENASA, 07 March 2006, 2 F; 77) Huallaga-Central, Tingo Maria, Naranjillo, 06 March 2006, SENASA, 1 F. 78) **Ica**: Ica, Bajo, Santiago, 06 December 2006, SENASA, 1 F. 79) **Junín**: Chincana, 29 January 2007, SENASA, 9 F. 80) **Lambayeque**: 2000, SENASA, 2 F. 81) **Libertad**: Santa Catalina, Laredo, Galindo, 18 May 2006, SENASA, 2 F; 82) Viru, Viru, Chanquin, 29 May 2006, SENASA, 2 F; 83) Jequetepeque, Guadalupe, La Cinta, 15 May 2006, SENASA, 2 F; 84) Santa Catalina, Laredo, Galindo, 15 June 2006, SENASA, 1 F; 85) Libertad: Santa Catalina, Laredo, La Merced, 07 June 2006, SENASA, 1 F; 86) Jequetepeque, Chepén, Talambo, 06 June 2006, SENASA, 3 F. 87) **Lima**: Asia, El Platanal, TML-fg, 16 March 1994, G.J. Steck & B. McPheron, 2 F; 88) La Molina, La Molina, lab colony, 18 March 1994, G.J. Steck & B. McPheron, 2 F; 89) lab colony in Lima SENASA, 5 F. 90) colony 34 in Vienna, Austria, 2012, IAEA-IPCL, 7 F. 91) **Piura**: San Lorenzo, Malingas, Canal Malingas Huaraguaos, 29 August 2006, SENASA, 3 F. 92) **Tumbes**: Zarumilla, Zarumilla Alta, Papayal, 28 February 2006, SENASA, 1 F; 93) Zarumilla, Zarumilla Alta, Papayal, 07 March 2006, SENASA, 1 F; 94) Zarumilla, Zarumilla Baja, La Palma, 05 April 2006, SENASA, 1 F. 95) **VRAE Region**: San Miguel, Qanan, Qanan, 24 August 2005, SENASA, 2 F; 96) San Miguel, Chaupin, Chaupin, 24 August 2005, SENASA, 3F; 97) 24 August 2005, SENASA, 5 F. **VENEZUELA**: 98) **Barinas**: Los Mesas, ex coffee, 1 M. 99) **Mérida**: Lagunilla, reared ex *Terminalia catappa*, 1989, A.L. Norrbom, 2 ?. 100) Mérida area, Finca San Antonio, >1600m, ex *Rubus glaucus* Benth., 31 May 1988, G.J. Steck & A.L. Norrbom, 1M 1?; 101) Merida, Sta. Rosa station, ca. 1600 m, ex coffee, 30 May 1988, G.J. Steck & A.L. Norrbom, 2 M. 102) **Vargas**: Los Caracas, Litoral central, sea level, reared ex *Terminalia cattapa*, 5 June 1988, C.J. Rosales, 1 M.

ITS1 in *Anastrepha* is highly base-biased with ~84–85% AT in the *fraterculus* species group. In addition, poly(A)/poly(T) subsequences of up to 20 or more bases are predominant. Initial PCR and Sanger sequencing of ITS1 resulted in what could be interpreted as substantial intra-individual polymorphism and/or PCR polymerase artifacts (slippage). Cloning of ITS1 PCR amplicons followed by comparative re-PCR/Sanger sequencing or direct Sanger sequencing of cloned DNA indicated that (1) PCR polymerase slippage-type artifacts were predominant (this does not rule out some level of intra-individual polymorphism) and (2) that the BigDye Terminator 3.1® (Thermo Fisher Scientific, Waltham, MA USA) sequence by synthesis (SBS) chemistry is highly resistant to polymerase slippage at this level of AT bias. In addition, it was found that less than perfect DNA quality can greatly increase polymerase artifacts.

A considerable effort was made to reduce PCR polymerase artifacts using high processivity polymerases, modified PCR conditions, and reaction adjuncts. The polymerases with the best performance included Phusion® (Thermo Fisher Scientific) under modified PCR conditions, KAPA® Hi Fi (KAPABiosystems, Wilmington, Massachusetts USA), and the KOD-based Platinum® Pfx (Thermo Fisher Scientific). No perfect solution was found; however, the overall level of PCR slippage in most non-degraded DNA samples was reduced to a point where confident analysis was possible. Homopolymer regions having greater than 14 or so A or T bases tended to exhibit PCR slippage even under optimal conditions, as also did sufficiently large repeats such as (ATT)_n_ with n>5. Since polymerase artifacts can originate from either PCR or replication, or both, it is possible, if not probable, that homopolymeric or repeat regions are exhibiting some level of intra-individual polymorphism. These regions, fortunately, tended to be localized as single isolated homopolymers or repeats towards the 5’ and/or 3’ ends of ITS1 in the *Anastrepha fraterculus* cryptic species complex allowing bidirectional resolution.

In addition, individual DNA samples were found to often react differently to PCR optimizations, particularly in the all too common situation of trapped specimens having a significant probability of degraded DNA. The use of high-salt PCR buffers with relatively high annealing temperatures was generally useful in these cases; however, PCR reaction adjuncts such as TMAC (tetra ammonium carbonate) used to preferentially improve AT bonding while significantly reducing polymerase slippage and artifacts in some cases often increased polymerase artifacts in other samples having the same ITS1 sequence. This latter effect generally occurred in cases of poor DNA quality. Resource limitations precluded PCR optimization for individual samples except in special cases. As a general strategy, KAPA® HiFi became the standard polymerase for PCR.

Primers utilized for PCR and sequencing are given in Table 1: ADL 18sF was based upon the 18s sequences NCBI EU179519 (*Anastrepha ludens*) and AF187101 (*Anastrepha fraterculus*); ADL 5.8sR was modified from CAS5p8sB1d (Ji et al. 2003); ADL ITS1 internal F and R were constructed from complete ITS1 sequences of *Anastrepha fraterculus* generated by direct sequencing using the above PCR primers. The primers internal to ITS1 allowed improved sequence quality for regions subject to polymerase slippage artifacts.

**Table 1.** Primers used for PCR amplification and sequencing of ITS1 in *Anastrepha fraterculus*.

Primer	Primer Sequence 5’–3’
ADL 18sF	TAA CTC GCA TTG ATT AAG TCC C
ADL 5.8sR	GAT ATG CGT TCA AAT GTC GAT G
ADL ITS1 internal F	GAT TGA ATG ATA AGT TAA TTT GTT CAC
ADL ITS1 internal R	GTT GCG AAT GTC TTA GTT CAA C

Primers supplied in lyophilized form by Integrated DNA Technologies (IDT) were reconstituted in 1X pH 8.0 Tris-EDTA (TE) (Thermo Fisher Scientific) to a 100µM stock solution. Working solutions were diluted in HyClone® nuclease-free water (Thermo Fisher Scientific) to 10µM for PCR amplification and/or 2.5µM for Sanger reactions. Experimentally, modified primers were synthesized incorporating 3’ phosphorothioate linkages to counter exonuclease degradation but this was not found to be helpful and was not continued.

Samples for DNA extraction generally consisted of 1 or more legs with attached muscle, or in some cases the complete thorax, frozen or in alcohol. The latter were air dried in a laminar flow hood to remove alcohol, then in both cases the samples were chopped, frozen in LN_2_, and powdered with a Mini-Beadbeater-96 (Biospec Products, Inc., Bartlesville, Oklahoma USA) using a glass bead. DNA extraction and cleanup utilized the DNeasy Blood and Tissue Kit ® (QIAGEN, Venlo, Netherlands) following the manufacturer's protocol with overnight digestion by Proteinase K using the supplied ATL buffer. Final elution was in 50µL of AE buffer.

PCR amplifications were carried using a GeneAmp® PCR System 9700 thermal cycler (Thermo Fisher Scientific) with the temperature program recommended by KAPA Biosystems for the KAPA HiFi hotstart polymerase: initial denaturation 95 °C/2 min followed by denaturation 98 °C/20 sec, annealing at 65 °C/15 sec, and extension at 72 °C/15 sec for 30 cycles with no final extension. PCR reactions also followed manufacturer's recommendations with a total volume of 20 µL, 0.3 µM final primer concentration, and 1U of polymerase. Mineral oil overlays (10 µL) were used. Visualization of amplification products was by planar gel electrochromatography on a 2–2.5% agarose gel and TAE buffer. Gels were stained using SYBR Safe (Thermo Fisher Scientific). If the PCR amplicons were deemed acceptable, then the PCR reactions were cleaned up by spin column using the Roche High Pure PCR Product Purification Kit (Roche Diagnostics Corporation, Indianapolis, Indiana USA) following the manufacturer's recommended protocol and the DNA concentration quantified by a NanoDrop® (Thermo Fisher Scientific) µv spectrophotometer prior to Sanger reaction assembly.

The Sanger sequencing utilized the BigDye® Terminator™ 3.1 chemistry (Thermo Fisher Scientific) following the manufacturer's recommendations for a 0.25X reaction mix and thermocycler temperature program. Sanger reaction products were cleaned of unincorporated dye terminators using the BigDye® Xterminator™ Purification Kit (Thermo Fisher Scientific) with a modified protocol using PCR tubes rather than a 96-well plate. Separation of Sanger reaction products and visualization utilized an Applied Biosystems 3100-Avant Genetic Analyzer (Thermo Fisher Scientific) upgraded to 3130 specifications.

Sequence Scanner v1.0 (Thermo Fisher Scientific) was used for base calling of raw sequences with visual interpretation for final decisions. Base editing and manual alignment, and cluster analysis utilized MEGA6: Molecular Evolutionary Genetics Analysis software version 6.01 (Tamura et al. 2013). Overall similarity between sequences was inferred by UPGMA (unweighted pair group method with arithmetic mean) cluster analysis (Sneath 1973) with distances computed by the maximum composite likelihood method (Tamura et al. 2004) in number of base substitutions per site with gaps eliminated.

Given the high variability in DNA quality, even between individual specimens from the same collection, a triage-type sequencing strategy evolved in which individual samples were evaluated at multiple stages of the analysis and rejected if they failed to meet certain experimentally determined criteria. This included extracted DNA concentration as determined by µv spectroscopy, visual intensity of amplicons from high stringency PCR reaction conditions, and initial single primer reverse strand sequencing of the r5.8s–r18s region. Only approximately 1/2 of the DNA from trapped specimens resulted in acceptable ITS1 sequence quality.

A subset of, or all, samples from each locality having the best DNA quality were sequenced using the full set of four primers to increase coverage and to allow bi-directional coverage of the regions showing possible intra-individual polymorphism and/or PCR slippage artifacts. Generally in these regions a consistent dominant sequence was present and could be reconstructed with bi-directional coverage and/or manual deconvolution of the overlapping sequences. The accuracy of this approach was verified during method development by direct sequencing of cloned PCR products.

## Results

**Figure 2.**
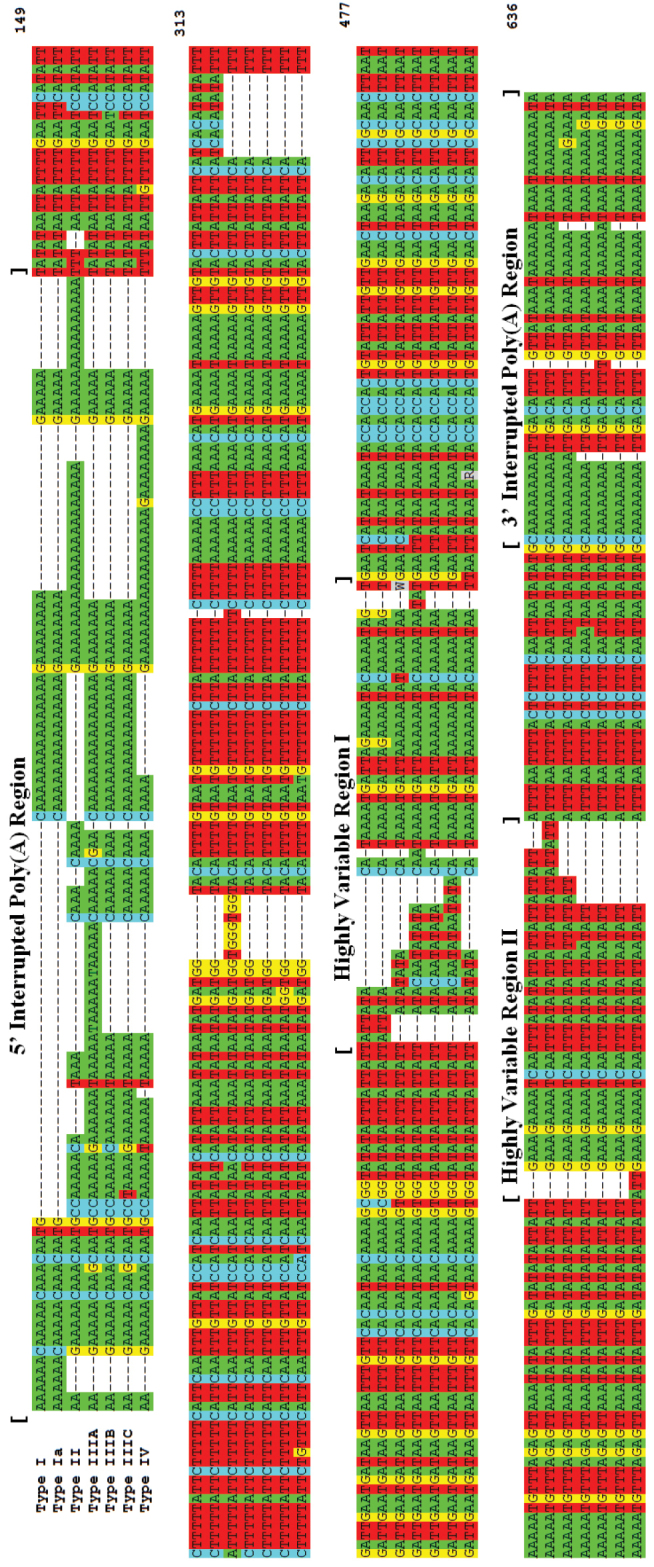
ITS1 polymorphic region sequences for Andean *Anastrepha fraterculus*; hypothetical alignment.

The polymorphic ITS1 region of the Andean *Anastrepha fraterculus* cryptic species complex ranges from 534–563 nucleotides (nt) in length and is significantly AT-biased at approximately 84% AT (Figure 2, all base numbers with respect to the hypothetical alignment in this figure). In this group, ITS1 can be considered as a mix of A/T homopolymers of variable length with intervening base interruptions including G and/or C and bounded by 5’ and 3’ interrupted poly(A) subregions 52–89nt (bases 1–123) and 44–46nt (bases 588–637) in length, respectively, having individual A homopolymers up to 22 bases. At the 3’ end of the 5’ poly(A) region (bases ~125–150) is a small variable region.

The majority of sequence polymorphism in the intervening subregion between the bounding interrupted poly(A) regions is concentrated in 2 highly variable sequence regions, here designated HRV I (bases 369–418) and HRV II (bases 516–556) respectively (Figure 2). These regions are polymorphic in sequence within the *Anastrepha fraterculus* cryptic species complex with respect to single nucleotide polymorphisms (SNPs) as well as in the lengths of repeats and/or progressive repeats. Within the relatively conserved regions between HRV I, HRV II, and the bounding poly(A) regions are occasional scattered SNPs as well as 2 unique expansions or insertions.

**Figure 3.**
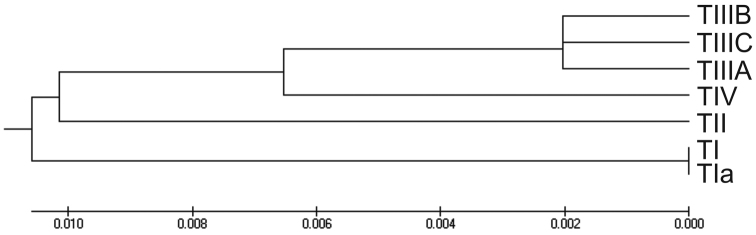
Overall similarity inferred by UPGMA (unweighted pair group method with arithmetic mean) cluster analysis (Sneath and Sokal (1973) of Andean *Anastrepha fraterculus* ITS1 sequence types (489nt). Distances were computed by the maximum composite likelihood method (Tamura et al. (2004) in number of base substitutions per site with gaps eliminated.

The ITS1 sequences of the Andean *Anastrepha fraterculus* cryptic species complex can be placed into 4 groups, here designated as ITS1 sequence types TI, TII, TIII, and TIV, with a variant of TI designated TIa and TIII further subdivided into 3 subtypes designated TIIIA, TIIIB, and TIIIC (Table 2). Representative sequences have been deposited in the NCBI. The pattern of overall similarity among these sequence types is visualized by UPGMA clustering (Figure 3).

**Table 2.** Results: ITS1 sequence types by country and collection.

ITS1 Sequence Type	Country	Collection number	NCBI Accession
**TI/TIa**	Argentina	1 (TI, TIa)	
	Bolivia	2 (TI, TIa), 3 (TI, TIa), 4 (TI), 5 (TI), 6, (TI), 7 (TI, TIa)	KT893864
	Brazil	8–16 (all TI)	
	Peru	62 (TI, TIa), 63 (TIa), 64 (TI), 65 (TI), 66 (TI), 68 (TI), 70 (TI), 95 (TI, TIa), 96 (TIa) 97 (TI, TIa)	KT893865
	Colombia	29–32, 39, 43–44	
	Costa Rica	47	
**TII**	Guatemala	52–53	
	Mexico	54–55	
	Venezuela	102	
	Colombia	33–37	
**TIIIA**	Ecuador	49, 51	KT893866
	Peru	56–61, 67, 71–94	KT893867
	Colombia	21	
**TIIIB**	Ecuador	50	
	Venezuela	100–101	
	Colombia	45	KT893868
**TIIIC**	Ecuador	48	
	Venezuela	99, 100	
	Colombia	17–20, 22–28, 38, 40–42, 45–46	KT893869
**TIV**	Ecuador	50	KT893870
	Peru	69	
	Venezuela	98, 101	

The ITS1 sequence type TI has a polymorphic region of length 534 or 537nt and is characterized by a unique CA to CATCACATATA expansion located approximately 40nt from the 5’ end of HVRI (bases 301 to 309), a 5’ poly(A) region unique within this complex, a unique repeat expansion (ATT)_2_ to (ATT)_3_ starting at base 363, and unique SNPs at bases 142, 351, 401, and 415. Inter-individual polymorphism is present in an (ATT)_n_ repeat with n=5 or 6 (the latter designated TIa in Figure 2) within HVRII starting at base 542 in the alignment.

**Figure 4.**
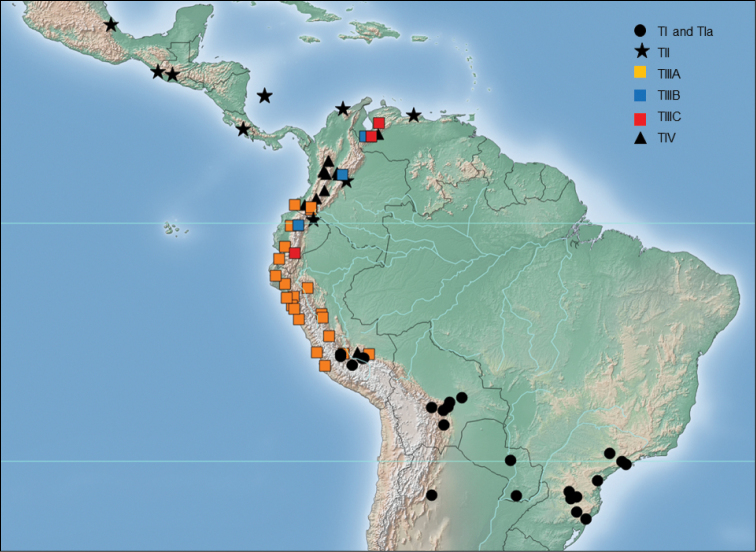
Geographical distribution of Andean *Anastrepha fraterculus* ITS1 sequence types.

The geographical distribution of TI in the central Andean region ranges from the Chiquitano forests into the eastern Andean dry valleys to at least 2000 m in Bolivia north to the higher elevation eastern dry valleys of the Cusco-Ayacucho region of Peru above *ca.* 2800 m. This ITS1 sequence type also extends south to Argentina (Tucumán), and east to Paraguay (Lopes 2010, Lopes et al. 2013) and at least southern and southeastern Brazil (Figure 4).

Geographical variation in the HVRII (ATT)_n_ repeat may be present with the n=5 variant (TI) most common in SE Brazil and n=6 (TIa) predominant in the Peru dry valleys; however, sample sizes are insufficient to determine if this is a real trend.

The ITS1 sequence type TII polymorphic region is 554nt in overall length and characterized by a unique expansion of TGG to TGGTGGGTGG starting at base 214, located 86nt from the 3’ end of the 5’ poly(A) region. The 5’ poly(A) region is unique in sequence within the *Anastrepha fraterculus* complex; however, the presence of consecutive A_22_ and A_15_ homopolymers (estimated lengths) increases the likelihood of PCR artifacts. The putative A deletions of TATATA to TTT at the 3’ end of this region is consistent but must be considered hypothetical. Additional unique ITS1 sequence elements includes a possible (T)_6_ to (T)_7_ expansion starting at base 251, SNPs at bases 149,191, 408, and 630, and the (ATT)_4_ repeat starting at base 539 in HVRII. An inter-individual polymorphism (A or T) was observed at base 418 and is indicated by the IUPAC ambiguity code W in the alignment.

The geographical distribution of TII in this study was restricted to the periphery of the northern Andes including Caribbean coastal sites of Colombia and Venezuela, and south along the eastern foothills of the Cordillera Oriental of Colombia to at least the Ecuador border (Figure 4). This sequence variant also characterizes the specimens from Mexico, Guatemala, Costa Rica, and the Isla de San Andrés (Colombia).

The ITS1 sequence type TIII polymorphic region ranged from 550-563nt in length. The three sequence subvariants in this group are very close in overall ITS1 similarity (Figure 3) but polymorphic in HVRI with a common CAATATA sequence starting at base 375. The geographical distribution of TIII was restricted to the central and northern Andean region (Figure 4).

Subtype TIIIA with the ITS1 polymorphic region of length 563nt, is the most divergent of the three with a TAAAAA to (TAAAAA)_3_ expansion starting at base 42 in the 5’ poly(A) region, unique insertions in HVRI including an A (base 389) and TA (at bases 416 to 417), and a possible A expansion at base 577.

This sequence subtype characterizes *Anastrepha fraterculus* of the lower elevations of the Andean periphery in the Pacific plain and Andean foothills and valleys of Peru (generally less than ~1200 m in elevation), from Ica in the south (eradicated by SENASA south of Lima?) through western Ecuador to the north into at least SW Colombia. Specimens with this sequence were also seen from the eastern Andean valleys of Peru from the Kosñipata Valley of Cusco to at least Amazonas (Figure 4).

The ITS1 sequence subtypes IIIB (length of polymorphic region 550nt) and IIIC (length 551nt) are very similar differing by 2 SNPs in the 5’ poly(A) region, an SNP at base 210, a complex expansion ATA to AATATA in HVRI starting at base 381, and putative homopolymer length polymorphisms towards the 3’ end of the ITS1 polymorphic region (bases 606 and 621).

The geographical distributions of these subtypes overlap with scattered collection localities from the Cordillera de Mérida in Venezuela south to the Andes of Ecuador (Figure 4) at elevations above ~1200 m. Specimens having these sequences were collected as larvae; so far no trapped adults have been seen. Larvae having the subtype IIIB sequence were collected from coffee (*Coffea* L. sp., Colombia) and toronja blanca, or pomelo (*Citrus maxima* (Burm.) Merr., Ecuador), and together with IIIC from Andean blackberry, (*Rubus glaucus* Benth.) in Venezuela.

The ITS1 sequence type TIV polymorphic region length is 553nt. This type is rather more generalized in ITS1 sequence within the *Anastrepha fraterculus* cryptic species complex, perhaps closer to the *fraterculus* species group groundplan than the others. Type TIV is characterized by a unique G interruption at base 99 in the 5’ poly(A) region, unique SNPs at bases 133, 160, 341, and 419, and by a unique repeat expansion (ATT)_2_ to (ATT)_3_ in HVRII starting at base 510.

The geographical distribution of TIV in the northern Andes extends from the Cordillera de Mérida of Venezuela, the Cordilleras Central and Oriental of Colombia, south to at least the northern Andes of Ecuador (Figure 4) generally above ~900 to 1000 m. In addition, specimens of *Anastrepha fraterculus* with this ITS1 sequence were collected in the vicinity of Echarate, in the southeastern Andean foothills of Peru at about 960 to 1400 m.

## Discussion

The ITS1 sequence types were not randomly distributed and they present a geographical pattern that is generally consistent with previous concepts about the *Anastrepha fraterculus* cryptic species complex of the Andean region.

ITS1 sequence type TI is widespread in South America from at least SE Brazil west into northern Argentina and north in the eastern Andes to at least the Cusco region of Perú. Specimens having this ITS1 sequence type include those from the IAEA, IPCL colonies originating from Argentina (Tucumán, sampled 2014 and 2015) and Brazil (Vacaria, sampled 2010, and Piracicaba sampled 2014, 2015), as well as the published sequences of specimens from Paraguay (Lopes 2010, Lopes et al. 2013). Concurrently, this type seems identifiable with *Anastrepha* sp. 1 (Selivon et al. 2004) (= *Anastrepha* sp. 1 aff. *fraterculus* Yamada & Selivon 2001, Selivon et al. 2005) and morphotype 1 of Hernández-Ortiz et al. (2012).

Specimens of the *Anastrepha fraterculus* complex having ITS1 sequence type TII were restricted to Mesoamerica and northeastern South America and include specimens from the IAEA, IPCL, colony originating from México (Xalapa, sampled 2013). A specimen with this ITS1 sequence originated from the los Caracas, Venezuela collection (1988) on which the “Vz-Lowland” population of Steck (1991) was based. TII appears to correspond to the “Mexican” form of Hernández-Ortiz et al. (2012), and likely includes specimens from “lowland” Venezuela. The latter were apparently not compared with the Mesoamerican *Anastrepha fraterculus* by Hernández-Ortiz et al. (2012).

The identity of the group having the ITS1 sequence type TIII is more complicated. Subtype TIIIA appears to be well defined and includes specimens from the IAEA IPCL, Perú colony (sampled 2014) and can probably be identified with the *Anastrepha* sp. 4 aff. *fraterculus* of Selivon et al. (2004) and the Peruvian morphotype of Hernández-Ortiz et al. (2012). *Anastrepha fraterculus* having the TIIIB and TIIIC sequence subtypes, however, are of uncertain taxonomic status and identity. Specimens from the highland Venezuela *Anastrepha fraterculus* collections of Steck (1991) include those with TIIIB, TIIIC, and TIV ITS1 sequences; unfortunately, specimens analyzed in the earlier isozyme analysis were destroyed during the process preventing molecular analysis of the same individuals. In Colombia, both subtype IIIB and type IV ITS1 sequences were found in specimens reared from larvae collected from coffee near Bogotá (this project); specimens from an IAEA, IPCL colony from Tolima, Colombia (sampled 2015) include subtype TIIIC. Both subtypes and type IV are present in collections of *Anastrepha fraterculus* from the Andes of Ecuador as well. It is not clear if a polymorphic ITS1 species is present in the highlands of the northern Andes, or if multiple sympatric species exist. Only specimens having ITS1 sequence type TIV were seen in trap samples (ICA) from Colombia, as well as from the IAEA, IPCL colony from Colombia (Ibagué, sampled 2014), the latter now lost.

Specimens having the TIV ITS1 sequence pattern seem to fit the “Andean” form of Hernández-Ortiz et al. (2012), but it remains unclear how those with the TIIIB and TIIIC sequence types fit into this morphotypic scheme. Steck (1991) failed to find isozyme heterogenity in the same collections of *Anastrepha fraterculus* from the Cordillera de Mérida, Venezuela, having specimens with the TIIIB, TIIIC, and TIV ITS1 sequence types. This is consistent with ITS1 sequence polymorphism. Additional collections from this region will be required to clarify this.

These results indicate that ITS1 sequence patterns can help resolve taxonomic structure in the *Anastrepha fraterculus* cryptic species complex, at least at some level. It remains to determine the lower limits of this resolution, ie. if a finer level of taxonomic structure in *Anastrepha fraterculus* exists in the Andean region. In addition, the sampling here was not geographically exhaustive. Significant parts of the Andean region remain poorly sampled including, but not restricted to, Ecuador and Venezuela. Beyond the Andean region; moreover, the diversity of the *Anastrepha* fauna in general and *Anastrepha fraterculus* in particular, of Amazonia and Guayana remain poorly known.
